# Modulation of Egg Elemental Metabolomics by Dietary Supplementation with Flavonoids and Orange Pulp (*Citrus sinensis*)

**DOI:** 10.3390/antiox14101179

**Published:** 2025-09-26

**Authors:** Evangelos Zoidis, Athanasios C. Pappas, Michael Goliomytis, Panagiotis E. Simitzis, Kyriaki Sotirakoglou, Savvina Tavrizelou, George P. Danezis, Constantinos A. Georgiou

**Affiliations:** 1 Laboratory of Nutritional Physiology and Feeding, Department of Animal Science, Agricultural University of Athens, 11855 Athens, Greece; ezoidis@aua.gr (E.Z.); stud217094@aua.gr (S.T.); 2 Laboratory of Animal Breeding and Husbandry, Department of Animal Science, Agricultural University of Athens, 11855 Athens, Greece; mgolio@aua.gr (M.G.); pansimitzis@aua.gr (P.E.S.); 3 Laboratory of Mathematics and Statistics, Department of Natural Resources Management and Agricultural Engineering, Agricultural University of Athens, 11855 Athens, Greece; sotirakoglou@aua.gr; 4 Chemistry Laboratory, Department of Food Science and Human Nutrition, Agricultural University of Athens, 11855 Athens, Greece; gdanezis@aua.gr (G.P.D.); cag@aua.gr (C.A.G.); 5 FoodOmics.GR Research Infrastructure, Agricultural University of Athens, 11855 Athens, Greece

**Keywords:** discriminant analysis, flavonoids, hen, hesperidin, ICP-MS, naringin, orange pulp, principal component analysis

## Abstract

Dried orange pulp (*Citrus sinensis*) is known for its antioxidant properties. This study aimed to examine the effects of adding dried orange pulp (OP) to the layers’ diets on the concentration of selected elements in the egg. The present work was part of a bigger project aiming to investigate the effect of orange pulp in layers’ diets on the performance of birds and egg quality. There were three dietary treatments and 63 layers per treatment, with 189 layers in total. Cages were the experimental units, and seven cages were allocated per treatment (n = 7). The dietary treatments were (1) a control treatment (C) that involved a basal diet without orange pulp addition, (2) an OP treatment with the addition of 9% dried orange pulp, and (3) a hesperidin–naringin (EN) treatment with 0.767 g hesperidin and 0.002 g naringin added per kg of diet; these levels of hesperidin and naringin represent those present in dried orange pulp for the OP treatment. Birds were fed the diets for 30 days. The diets had similar energy and protein levels and contained the same vitamin and mineral premixes. The analyzed egg (yolk, albumen, shell) elemental profile consisted of As, Ca, Cd, Co, Cr, Cu, Fe, Mg, Mn, Ni, Sb, Se, Sr, V, and Zn and was determined via Inductively Coupled Plasma Mass Spectrometry (ICP-MS). Dried orange pulp supplementation significantly altered the elemental profile. OP largely altered the element concentrations in albumen and egg yolk. Most notably, it decreased the concentrations of Co (*p* < 0.001), Fe (*p* < 0.001), Mn (*p* < 0.001), Ni (*p* = 0.046), and Se (*p* = 0.035) in egg yolk and those of Co (*p* = 0.011), Fe (*p* = 0.025), Cr (*p* = 0.049), Cu (*p* = 0.001), and Se (*p* = 0.014) in albumen. In addition, it decreased the concentrations of As (*p* = 0.025) and Ca (*p* = 0.025) in the eggshell. Principal component analysis was applied to the concentrations of the examined elements in all egg parts to explore the relationships between the elements and detect those capable of distinguishing samples, resulting in the apparent separation of yolk, albumen, and eggshell samples. Further analysis revealed that all samples were clustered into the three dietary treatments, resulting in 100% correct classification. The chelating and antioxidant capacities of flavonoids are intricate and rely on a variety of factors. OP supplementation modulated the deposition of specific elements in egg parts in comparison to those from layers fed a typical diet. Thus, this study indicated that eggs with specialized elemental profiles could be created.

## 1. Introduction

The term “biometals” refers to trace metals that are involved in regular body functions [1], and the totality of metal and metalloid species found is referred to as the “metallome” [2]. Numerous elements in the organism interact and compete during absorption, metabolism, and homeostasis. Thus, measuring and monitoring these elements in samples are the main goals of elemental metabolomics. The application of elemental metabolomics as a methodology in agriculture, nutrition, and food science has emerged in the last decade [3,4]. Moreover, consumer demand for premium-quality foods has led to the development of functional foods and designer eggs [5], which consist of, but are not restricted to, lower-cholesterol eggs, ω-3-enriched eggs, and eggs enhanced with minerals, pigments, vitamin E, and a variety of bioactive substances [6,7,8,9,10].

Flavonoids represent a group of bioactive polyphenols known as plant-derived secondary metabolites, abundant in vegetables and fruits [11]. They participate in the defensive systems of antioxidants since they have free radical scavenging and metal chelating functions [12,13].

Recently, the rising costs of feedstuffs such as cereal grains and soybean products have steadily increased the expenses associated with livestock production. Therefore, low-input feeding techniques that rely on alternative food supplies, including agro-industrial byproducts, are necessary to reduce animal nutrition costs [14]. Moreover, animal products must shield consumers from oxidative stress-related degenerative disorders in addition to meeting nutritional needs. To promote mental and physical health, consuming foods rich in functional components (i.e., nutraceuticals) can corroborate the body’s defenses against these disorders [15,16].

The use of agro-industrial byproducts in livestock nutrition reduces the environmental impact of production, improves the scope of the circular economy, and promotes sustainability within the food chain of ingredients with added value. These byproducts are thought to be an inexpensive source of certain nutrients [17]. Citrus pulp, a byproduct rich in antioxidant flavonoids, has already been utilized in poultry diets, with diverse results in terms of performance output but significantly positive effects on antioxidant properties. The use of 2% dried orange pulp [18] or 80–480 mg/kg of dried tangerine peel extract [19] had a positive effect on performance parameters, but when 5–10% citrus pulp was provided, growth parameters decreased [20]. Furthermore, flavonoids, such as those present in citrus byproducts, have been examined to assess their potent beneficial effects on poultry, since they are regarded as natural antioxidants and accepted as safer than synthetic ones [21,22]. Most notably, the inclusion of hesperidin [23,24] or naringin [25] in chickens’ diets has improved growth performance and antioxidant capacity.

This study was part of a larger project aiming to utilize agro-industrial byproducts, more specifically citrus byproducts, in poultry diets and, thus, promote sustainability and meet consumer demands for products rich in natural antioxidants. In detail, the wider project aimed to include dried orange pulp, rich in antioxidants, in layers’ diets and examine the performance of birds and egg quality. According to our knowledge, no data exists regarding the effect of adding orange pulp to hens’ diets on the egg metallome. This study envisaged how eggs with an altered metallome would satisfy the nutritional demand of consumers and protect them from metabolism-related issues. Taking into account the fact that some elements (i) act as structural components (Calcium-Ca); (ii) participate in activation or/and signaling (Magnesium-Mg, Ca); (iii) are components of enzymes, proteins, and hormones (Cobalt-Co, Chromium-Cr, Copper-Cu, Iron-Fe, Manganese-Mn, Nickel-Ni, Selenium-Se, Vanadium-V and Zinc-Zn); (iv) are related to toxicity (Arsenic-As and Cadmium-Cd); (v) or have an undefined role (Antimony-Sb and Strontium-Sr), fifteen elements, namely As, Ca, Cd, Co, Cr, Cu, Fe, Mg, Mn, Ni, Sb, Se, Sr, V, and Zn, were detected in the egg.

## 2. Materials and Methods

### 2.1. Trial Design and Experimental Procedures

This study was part of a larger project aiming to investigate the role of adding orange pulp to layers’ diets, and the methods used in our experiment were approved by the Research Ethics Committee of the Department of Animal Science of the Agricultural University of Athens [26]. Data on the nutritional requirements, feed ingredients, chemical composition, and the performance of birds have been presented previously [26]. In brief, 189 45-wks old Lohmann Brown-Classic hens were placed into 3 treatment groups. For each treatment group, there were 7 replicate cages. The cage was the experimental unit; thus, n = 7. There were 9 hens per cage, with 63 per treatment. Regarding treatments, (1) hens receiving the control treatment (C) were offered a basal diet without added orange pulp, (2) those receiving dried orange pulp (OP) treatment were offered a diet with 9% dried orange pulp, and (3) hens receiving hesperidin–naringin (EN) treatment were offered a diet with 0.767 g/kg of hesperidin (Tokyo Chemical Industry Co., Ltd., Tokyo, Japan) and 0.002 g/kg of naringin (Alfa Aesar GmbH & Co KG, Emmerich am Rhein, Germany). OP contained peels and seeds and originated from leftovers from the processing of the oranges. The inclusion of 9% OP in the experimental diet was based on (i) earlier findings from our research team [26,27] reporting that adding 0.75 g/kg of hesperidin to the diet improved yolk oxidative stability; (ii) a chemical analysis that revealed hesperidin and naringin contents in OP of 8.52 and 0.0223 g/kg, respectively [28,29,30]; and (iii) ration calculations performed to meet hens’ nutrient requirements [31]. Thus, with a 9% inclusion level, the hesperidin and naringin contents in the OP diet were 0.767 g/kg (8.52 × 0.09) and 0.002 g/kg (0.0223 × 0.09), respectively. Since the inclusion of 9% orange pulp provided the aforementioned levels of hesperidin and naringin, EN treatment was designed to represent those levels present in dried orange pulp compared to OP treatment. During the design of this study, the EN treatment was included to explore whether the anticipated results could be attributed to hesperidin and naringin solely or to other compounds present in the OP. The trial lasted for 30 days, and hens were fed diets with similar energy and protein contents. The OP proximate analysis revealed dry matter of 951.3 g/kg, crude protein of 58.3 g/kg, fat content of 12 g/kg, and fiber of 101 g/kg [26]. Feed was given ad libitum. Birds had free access to water. The light/dark program included 16 h of continuous light and 8 h of darkness. On the 30th day of the experiment, 3 eggs per replicate cage were collected, with 21 per treatment, or 63 in total, and subjected to elemental analysis.

### 2.2. Determination of Selected Elements in Eggs

Inductively Coupled Plasma Mass Spectrometry (Perkin Elmer, Elan 9000, SCIEX, Toronto, ON, Canada) was applied to determine the elemental profiles of eggs [7,8]. Suprapur^®^ 65% *w*/*v* nitric acid was used (Merck, Darmstadt, Germany). Multi-element standards were provided by Inorganic Ventures, Christiansburg, VA, USA. Ultrapure water with 18.2 MΩ cm^−1^ resistance (Merck Millipore, Darmstadt, Germany) was used. A microwave-assisted digestion system (CEM, Mars X-Press, Matthews, NC, USA) was used for sample digestion. Wet samples (1 g yolk, 1 g albumen, and 0.1 g of eggshell) were weighed and placed in a polypropylene tube. The predigestion of samples occurred for 30 min. The resulting sample suspensions were transferred quantitatively into the polytetrafluoroethylene MARS vessels (CEM, Mars X-Press, Matthews, NC, USA). The MARS digestion program included ramping for 20 min from 100 W to 1200 W and stability for 15 min. The maximum temperature was 200 °C, and then 15 min of cool-down followed. No sample losses occurred since the polytetrafluoroethylene vessels were sealed. Sample solutions were filtered with 0.20 μm/15 mm filters (Chromafil, Macherey-Nagel, Düren, Germany). Sample solutions were diluted with ultrapure water and injected into the ICP-MS tool. ICP-MS was performed with a nebulizer gas flow of 0.91 L min^−1^, a lens voltage of 8.5 V, an ICP RF (radiofrequency) power of 1050 W, and a pulse stage voltage of 950 V. High-purity standards were used for the calibration curves.

### 2.3. Statistical Analysis

The Statgraphics statistical package (version 17) was used. Data are shown as least squares means ± standard errors. Data distribution was explored with the Shapiro–Wilk test and graphical methods (Q–Q plots) to assess normality. The homogeneity of variances was checked using Levene’s test. The effects of dietary treatments on element concentrations were explored using one-way analysis of variance (ANOVA). When appropriate, post hoc analyses were performed using Duncan’s multiple range test. When the assumptions of ANOVA, such as the normality and homogeneity of variances, were not met, a nonparametric test (Kruskal–Wallis) was performed, followed by Dunn’s multiple range test. A reduction in data dimensionality and an investigation of the relationships between the elements were performed with principal component analysis (PCA). Discriminant analysis was performed on albumen, yolk, or shell data to investigate the differentiation of samples by dietary treatment and further explore the elements’ ability to distinguish the samples. Discriminant variables were selected using Wilk’s lambda (λ) criterion. For all tests, the significance level was set at 5%.

## 3. Results

### 3.1. Elemental Content

The concentrations of selected elements in egg albumen are presented in Table 1. In our experiment, the inclusion of 9% dried orange pulp decreased the concentrations of Co (39%), Cr (15%), Cu (52%), Fe (15%), and Se (63%) compared to the control treatment. Hesperidin and naringin supplementation significantly decreased the concentrations of Co (24%) and Ni (33%) and increased that of Sb (60%) compared to the control (Table 1).

The concentrations of selected elements in egg yolk are illustrated in Table 2. The supplementation of dried orange pulp (*Citrus sinensis*) significantly decreased levels of Co (19%), Fe (20%), Mn (41%), Ni (12%), and Se (58%), while increasing the concentration of V (12%), compared to the control.

Moreover, the supplementation of feed with hesperidin plus naringin at 0.767 and 0.002 g/kg notably decreased, compared to the control, the concentrations of As (15%), Co (13%), and Se (50%) (Table 2).

Concentrations of selected elements in eggshell are presented in Table 3. The supplementation of 9% dried orange pulp significantly decreased the concentrations, compared to the control, of As (3.5-fold), Ca (7%), Cd (5-fold), Cr (12%), Cu (4.3-fold), and V (35%). Adding Hesperidin and naringin to the diet decreased, compared to the control, the concentrations of the elements Cr (15%), Cu (3.8-fold), Mg (14%), Se (10-fold), Sr (9%), and V (26%) (Table 3).

### 3.2. Principal Component Analysis

**Figure 1:**
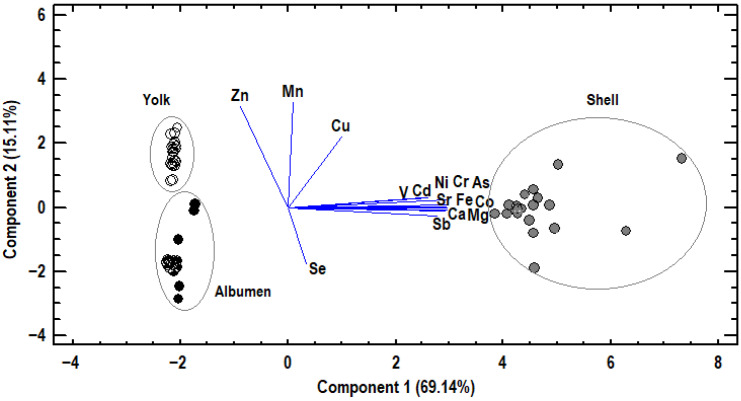
Principal components analysis. Plot of selected elements in albumen, yolk, or shell samples, as a function of the two first principal components.

Principal component analysis (PCA) was applied to elements deposited in egg albumen, yolk, and eggshell to examine relationships between elements and find those that were capable of differentiating the samples (Figure 1). Two principal components were found via PCA, which accounted for 84.24% of the total variability. In Figure 1, the selected elements in albumen, yolk, and eggshell samples are illustrated as a function of both the first and second principal components. In total, 69.14% of the total variability was explained by the first principal component (PC1), and this variability was defined by As, Ca, Cd, Co, Cr, Fe, Mg, Ni, Sb, Sr, and V. These elements were placed away from the axis’ origin and clustered together, indicating strong positive correlations. Samples selected from eggshells clustered near them and, therefore, had higher concentrations of these elements compared to the samples from egg yolk and egg albumen, clustered on the negative side of PC1. Moreover, 15.11% of total variability was explained by the second principal component (PC2) and defined by Cu, Mn, Se, and Zn. Cu, Mn, and Zn were located close together on the positive side of PC2, suggesting a positive correlation. These elements were positioned opposite to Se and, therefore, were negatively correlated. Samples collected from egg yolk were clustered near Zn, Mn, and Cu, indicating higher concentrations of these elements compared to those originating from egg albumen and eggshell. In contrast, albumen samples were clustered on the negative side of PC2, near Se, and they, therefore, had higher concentrations of Se. Consequently, it was possible to clearly distinguish between the albumen, yolk, and eggshell samples.

### 3.3. Discriminant Analysis

**Figure 2:**
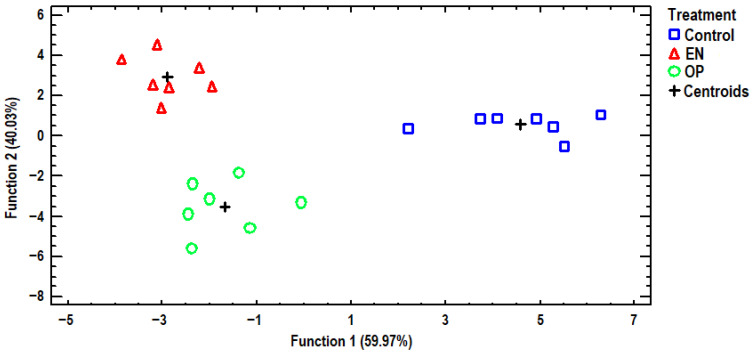
Discriminant plot separating egg albumen samples, according to dietary treatment, based on determined elements.

**Figure 3:**
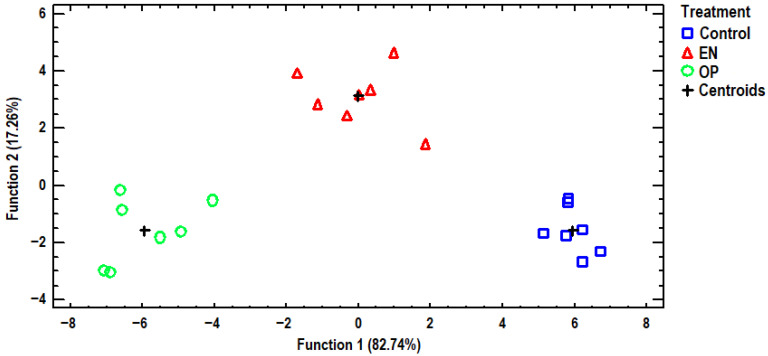
Discriminant plot separating egg yolk samples, according to dietary treatment, based on determined elements.

**Figure 4:**
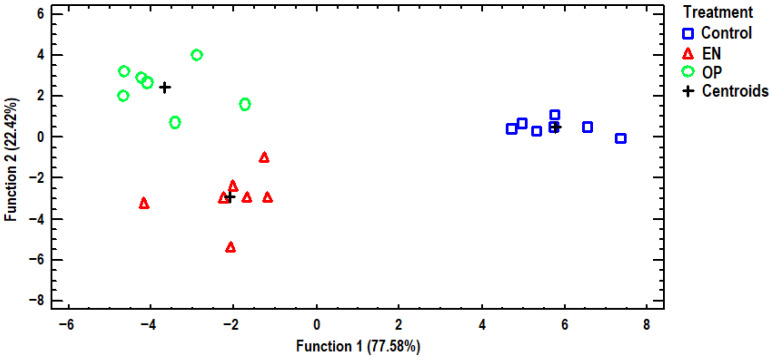
Discriminant plot separating eggshell samples, according to dietary treatment, based on determined elements.

Discriminant analysis was further employed to distinguish the data from selected elements of egg albumen to examine whether the samples could be differentiated against the three dietary treatments, as well as find those elements capable of distinguishing the samples. A discriminant plot of the first two discriminant functions, which successfully distinguishes the samples, can be seen in Figure 2. All examined samples were clustered by dietary treatment, and the classification was 100% accurate. The control group samples were placed on the positive side of the first discriminant function, far away from all the treated samples. Additionally, the second discriminant function distinguished between samples from treatments EN and OP. A stepwise discriminant analysis showed that Co, Cu, Ni, and Sb made the largest contributions to the observed differentiation. Another discriminant plot is presented in Figure 3, based on selected elements of egg yolk, discriminating samples according to dietary treatment. One discriminant function was statistically significant (*p* = 0.002) for differentiating the samples, and a 100% classification accuracy was achieved. The control group samples were clustered in the bottom right-hand corner of the plot, very distant from all the samples of the treatment groups. Conversely, samples from the OP treatment group were placed on the left side of the plot, furthest away from the samples of the control group, showing that the greatest discrimination for the selected elements was noted for the dietary treatment groups. Samples from the EN treatment group were placed in the middle of the plot, and accordingly, samples from the EN treatment group had intermediate contents of the examined elements. In addition, a stepwise discriminant analysis showed that Co, Cr, Mn, Se, and Sr were mainly responsible for distinguishing the observations. We attempted to further differentiate the samples based on the analyzed elements of the eggshell, as shown in Figure 4. In this instance, the control group’s samples were also grouped far away from the OP treatment group’s samples, as in the egg yolk’s discriminant plot, again indicating that the most discriminations in the examined elements were noted between these dietary treatments. One discriminant function was statistically significant (*p* = 0.004), and once again, a 100% classification accuracy was achieved. In addition, stepwise discriminant analysis showed that Ca, Co, Cu, and Sr contributed the most to the observed discrimination.

## 4. Discussion

### 4.1. Antioxidant Substances in Animals’ Nutrition

To the best of our knowledge, this is the first report to study the effects of supplementing laying hens’ diets with dried orange pulp and how flavonoids present in pulp can alter the egg metallome. The results of this study may support the use of agro-industrial byproducts, more specifically citrus byproducts, in poultry diets to meet consumer demands for products rich in natural antioxidants and to promote sustainability, since they are used elsewhere rather than discarded. Previously, the potential of orange pulp for improving the laying rate, egg quality, and oxidative stability was investigated, with promising results [32]. A crucial component of improving animal welfare and health, as well as livestock performance and output, is improving the composition of food [33]. This study, conducting research from a different perspective, envisaged how eggs with altered metallomes would satisfy the nutritional demands of consumers and protect them from metabolism-related issues.

To delay the natural deterioration and peroxidation of foods, the food industry frequently uses synthetic antioxidants and antimicrobial food additives [34]. Long-term use of these compounds has been associated with an increased risk of carcinogenesis, although it is thus far unclear exactly how they affect human health [35]. Therefore, it is crucial to identify natural substitutes for these ingredients that will not have any negative long-term health effects. Consuming animal products, such as meat and eggs, that are high in these healthy nutraceuticals can improve human health and immunity to illness [36,37,38].

After extracting orange fruit juice and drying the leftover material, dried orange pulp can be used as an industrial byproduct. Orange pulp, which is made of pulp, peel, and seeds, has naturally occurring active ingredients, such as flavonoids and phenolic acids [38,39]. In addition, orange peel contains pectin and dietary fiber. Dietary fiber is essential for preventing the development of cancer, diabetes, heart disease, and gastrointestinal issues [32]. Vitamin C and the flavonoids found in orange pulp have chelating, antibacterial, immune-stimulating, and beneficial antioxidant qualities [40].

Naringin and hesperidin, acting as potential chelating agents, can be used to modulate the egg metallome by changing the contents of specific elements relative to the contents of those elements in the eggs of hens receiving a control diet or a control diet augmented with vitamin E [7]. Orange pulp supplementation at a rate of 50 g/kg combined with organic selenium has recently been found to enhance the nutritional value and oxidative stability of meat without negatively impacting broiler chicken performance [41]. Adding up to 12% citrus pulp to laying hens’ diets has also been shown to have no detrimental effects on hens’ performance or egg quality [42]. This study was part of a larger project, and the performance results demonstrated that adding 90 g/kg of orange increased the oxidative stability of the laying hens’ eggs, though its supplementation was linked to negative outcomes for the laying hens’ performance and egg quality [26]. Reduced feed intake values in birds fed OP may be attributed to the low palatability of OP, and this factor may have affected the intake and availability of micro- and macronutrients in this research. Future studies on OP and elements should consider the fact that gradually increasing citrus pulp levels in layers’ diets may act as an adaptation and positively affect feed intake, overcoming any adverse effects on performance.

According to earlier research, quercetin, another flavonoid, protects erythrocytes from oxidative damage, while naringin and hesperidin exhibit neuroprotective effects linked to their metal chelating properties [43,44]. Additionally, citrus flavonoids may assist in modulating the concentrations of several minerals, according to a study that examined their role in the bioavailability of Ca, Fe, Mg, P, and Zn in eggshells, as well as their role in preventing bone loss [45].

### 4.2. “Metallophenolomics” and Alteration of Egg Metallome

Orange pulp has been shown to impact trace-element homeostasis in hen eggs, as evidenced by variations in the elemental profiles of the three egg parts.

Compared to the yolk and albumen, the eggshell had the highest concentrations of most elements in this study. Furthermore, the yolk contained larger amounts than the albumen, suggesting that the yolk stores fundamental elements, deposits the majority of egg minerals, and facilitates their efficient transmission to the growing embryo through the yolk sac [8]. Specialized proteins such as Cu transporters, Zn transporters, and Mg channels are responsible for maintaining mineral homeostasis, which is the balance between the absorption and excretion of elements [46].

Orange pulp supplementation decreased the concentrations of most elements in all egg components. In particular, the concentrations of Co, Cr, Cu, Fe, Sb, and Se were decreased in egg albumen; furthermore, Co, Fe, Mn, Ni, and Se concentrations were decreased in egg yolk, and As, Ca, Cd, Cr, Cu, Se, and V concentrations were decreased in eggshell. Only the content of V was increased in egg yolk with orange pulp supplementation compared to the control and EN-fed layers. Notably, elements including As, Cd, Cr, and Cu, thought to be toxic at high concentrations, were reduced following orange pulp supplementation. That said, it is difficult to reach definite conclusions regarding the mitigation of or increase in toxicity. Plant studies have shown that flavonoids are significant components influencing the tolerance of toxic elements such as Cd [47]. Furthermore, polyphenols have been demonstrated to protect cells from oxidative damage induced by excess Fe concentrations [48]. Some flavonoids have also been shown to reduce trace element absorption when added to a diet. Grape seed extracts, for example, have been shown to reduce Fe and Zn intestinal absorption in a dose-dependent manner [8]. Nonetheless, more investigation is needed to determine whether dietary orange pulp supplementation and metal complexing properties are directly related.

The formation of flavonoid complexes with elements could be linked to flavonoids’ effects on the homeostasis of trace elements. Recently, many flavonoids have attracted a lot of interest for use as nutritional additives [8]. Flavonoids are best known for their ability to scavenge free radicals. Their beneficial effects are connected to their ability to scavenge free radicals and exhibit complexation capacities with metal ions [49]. This study’s results are consistent with prior data on rodents, indicating that the production of chelates and complexes with organic ligands can affect the interaction between the absorption and membrane transport of certain metal ions and the tissue availability of several elements [50].

Flavonoids’ chelating and antioxidant properties in in vivo biological systems are complex and depend on varied conditions such as bioavailability at the target tissue site; relatively low absorption; metabolite production following absorption, which reduces their activity; and the surrounding flavonoid environment, which can greatly influence to what extent polyphenols exert their effects [51]. Variations in flavonoids may be attributed to their chelation sites, stereoisomers, and structures. Additional research is needed to determine the metal complexation properties of various orange pulp metabolite levels in eggs. In this context, the concept of “metallophenolomics” was recently proposed as a subgroup of metallomics for studying phenolics–metal(loid) complexations [52].

In this study, adding orange pulp to hens’ diets modulated concentrations of several elements in specific egg compartments. Various factors, such as dosage, the type of substance, and combination with other compounds, seem to impact trace element absorption and assimilation by polyphenols.

## 5. Conclusions

This study showed that orange pulp may modify the elemental profiles of eggs due to the existence of flavonoids that can change the amounts of specific elements present in egg parts. The observed results showed complicated interactions that cannot be solely attributed to element chelation, indicating that more research is required.

The future is promising regarding the use of orange pulp in this field, but more research is needed to understand the appropriate levels of orange pulp that should be added to diets to create eggs with altered metallomes without compromising bird performance. In addition, it is hypothesized that interactions could be addressed through feeding studies with fine-tuned elemental supplementation and concurrently differentially adjusted concentrations of flavonoids.

## Figures and Tables

**Table 1:** The effect of dietary supplementation with hesperidin plus naringin or dried orange pulp (*Citrus sinensis*) on selected elements concentrations in laying hens’ egg albumen (n = 7).

	Treatment				
Elements (μg/kg)	C	EN	OP	SEM	*p*-Value
As	5.04	4.79	4.64	0.147	0.178
Ca	6.78 × 10^4^	7.32 × 10^4^	4.99 × 10^4^	918 × 10	0.199
Cd	4.21	3.21	4.06	0.801	0.644
Co	2.49 ^b^	1.89 ^a^	1.77 ^a^	0.158	0.011
Cr	95.0 ^b^	85.1 ^ab^	80.9 ^a^	3.90	0.049
Cu	733 ^b^	168 × 10 ^b^	353 ^a^	317	0.001
Fe	584 × 10 ^b^	525 × 10 ^ab^	499 × 10 ^a^	206	0.025
Mg	1.10 × 10^5^	1.10 × 10^5^	9.53 × 10^4^	831 × 10	0.369
Mn	65.9	59.3	54.7	6.53	0.492
Ni	67.2 ^b^	38.1 ^a^	44.8 ^ab^	8.68	0.012
Sb	1.63 ^a^	2.61 ^b^	1.49 ^a^	0.229	0.005
Se	240 × 10 ^b^	354 × 10 ^b^	891 ^a^	774	0.014
Sr	158	142	150	11.5	0.628
V	64.9	66.8	66.2	1.08	0.463
Zn	113 × 10	130 × 10	107 × 10	92.6	0.225

^a,b^ means in a row sharing no common superscript differ significantly (*p* < 0.05).

**Table 2:** The effect of dietary supplementation with hesperidin plus naringin or dried orange pulp (*Citrus sinensis*) on selected elements concentrations in laying hens’ egg yolk (n = 7).

	Treatment				
Elements (μg/kg)	C	EN	OP	SEM	*p*-Value
As	4.24 ^b^	3.60 ^a^	3.94 ^ab^	0.199	0.048
Ca	1.07 × 10^6^	1.05 × 10^6^	9.59 × 10^5^	4.70 × 10^4^	0.227
Cd	4.86	4.43	4.51	0.242	0.434
Co	3.96 ^b^	3.43 ^a^	3.21 ^a^	0.118	<0.001
Cr	145	141	151	3.22	0.138
Cu	173 × 10	168 × 10	160 × 10	94.1	0.639
Fe	737 × 10^2 b^	676 × 10^2 b^	594 × 10^2 a^	212 × 10	<0.001
Mg	1.39 × 10^5^	1.41 × 10^5^	1.51 × 10^5^	541 × 10	0.299
Mn	125 × 10 ^b^	115 × 10 ^b^	748 ^a^	78.2	<0.001
Ni	74.9 ^b^	67.5 ^ab^	65.8 ^a^	2.85	0.046
Sb	1.41	1.43	1.20	0.128	0.387
Se	1286 ^b^	637 ^a^	536 ^a^	201.6	0.035
Sr	699	684	637	39.8	0.531
V	80.7 ^a^	84.7 ^ab^	90.3 ^b^	1.89	0.008
Zn	432 × 10^2^	420 × 10^2^	443 × 10^2^	154 × 10	0.594

^a,b^ means in a row sharing no common superscript differ significantly (*p* < 0.05).

**Table 3:** The effect of dietary supplementation with hesperidin plus naringin or dried orange pulp (*Citrus sinensis*) on selected elements concentrations in laying hens’ eggshell (n = 7).

	Treatment				
Elements (μg/kg)	C	EN	OP	SEM	*p*-Value
As	82.5 ^b^	40.8 ^ab^	23.1 ^a^	17.1	0.008
Ca	2.49 × 10^8 b^	2.37 × 10^8 ab^	2.31 × 10^8 a^	5.58 × 10^6^	0.049
Cd	342 ^b^	115 ^ab^	68.5 ^a^	88.2	0.041
Co	361	283	273	33.7	0.160
Cr	840 ^b^	719 ^a^	744 ^a^	22.5	0.003
Cu	640 × 10 ^b^	169 × 10 ^a^	149 × 10 ^a^	165 × 10	0.043
Fe	1.96 × 10^6^	1.86 × 10^6^	1.85 × 10^6^	5.45 × 10^4^	0.307
Mg	3.56 × 10^6 b^	3.06 × 10^6 a^	3.43 × 10^6 ab^	1.32 × 10^5^	0.038
Mn	939	600	535	137	0.245
Ni	913 × 10	879 × 10	928 × 10	359	0.618
Sb	67.0	48.4	64.4	7.71	0.211
Se	358 × 10 ^b^	362 ^a^	192 × 10 ^ab^	899	0.002
Sr	1.07 × 10^5 ab^	9.77 × 10^4 a^	1.14 × 10^5 b^	406 × 10	0.031
V	107 × 10 ^b^	794 ^a^	702 ^a^	74.8	0.007
Zn	11 8× 10^2^	107 × 10^2^	99 × 10^2^	109 × 10	0.491

^a,b^ means in a row sharing no common superscript differ significantly (*p* < 0.05).

## Data Availability

Data is contained within the article.

## Abbreviations

C	control group;
EN	hesperidin plus naringin group fed with 0.767 g hesperidin plus 0.002 g naringin per kg feed;
OP	orange pulp group fed with 90 g dried orange pulp per kg feed;
ICP-MS	inductively coupled plasma-mass spectrometry.

## References

[1] Silver S. (2011). BioMetals: A historical and personal perspective. Biometals.

[2] Mounicou S., Szpunar J., Lobinski R. (2009). Metallomics: The concept and methodology. Chem. Soc. Rev..

[3] Wishart D. (2008). Metabolomics: Applications to food science and nutrition research. Trends Food Sci. Technol..

[4] Wu W., Zhang L., Zheng X., Huang Q., Farag M.A., Zhu R., Zhao C. (2022). Emerging applications of metabolomics in food science and future trends. Food Chem. X.

[5] Alagawany M., Farag M.R., Dhama K., Patra A. (2018). Nutritional significance and health benefits of designer eggs. World’s Poult. Sci. J..

[6] Réhault-Godbert S., Guyot N., Nys Y. (2019). The Golden Egg: Nutritional Value, Bioactivities, and Emerging Benefits for Human Health. Nutrients.

[7] Pappas A.C., Zoidis E., Goliomytis M., Simitzis P.E., Sotirakoglou K., Charismiadou M.A., Nikitas C., Danezis G., Deligeorgis S.G., Georgiou C.A. (2019). Elemental Metabolomics: Modulation of Egg Metallome with Flavonoids, an Exploratory Study. Antioxidants.

[8] Zoidis E., Pappas A.C., Goliomytis M., Simitzis P.E., Sotirakoglou K., Tavrizelou S., Danezis G., Georgiou C.A. (2021). Quercetin and Egg Metallome. Antioxidants.

[9] Filipiak-Florkiewicz A., Dymińska-Czyż M., Szymczyk B., Franczyk-Żarów M., Kostogrys R., Florkiewicz A., Lukasiewicz M. (2024). Design of Physicochemical Properties of Eggs as a Result of Modification of the Fat Fraction of Laying Feed. Molecules.

[10] Usturoi M.G., Rațu R.N., Crivei I.C., Veleșcu I.D., Usturoi A., Stoica F., Radu Rusu R.-M. (2025). Unlocking the Power of Eggs: Nutritional Insights, Bioactive Compounds, and the Advantages of Omega-3 and Omega-6 Enriched Varieties. Agriculture.

[11] Roy A., Khan A., Ahmad I., Alghamdi S., Rajab B.S., Babalghith A.O., Alshahrani M.Y., Islam S., Islam M.R. (2022). Flavonoids a Bioactive Compound from Medicinal Plants and Its Therapeutic Applications. BioMed Res. Int..

[12] Hilal B., Khan M.M., Fariduddin Q. (2024). Recent advancements in deciphering the therapeutic properties of plant secondary metabolites: Phenolics, terpenes, and alkaloids. Plant Physiol. Biochem..

[13] Bolat E., Sarıtaş S., Duman H., Eker F., Akdaşçi E., Karav S., Witkowska A.M. (2024). Polyphenols: Secondary Metabolites with a Biological Impression. Nutrients.

[14] Vasta V., Luciano G. (2011). The effects of dietary consumption of plants secondary compounds on small ruminants’ products quality. Small Rumin. Res..

[15] Pisoschi A.M., Pop A. (2015). The role of antioxidants in the chemistry of oxidative stress: A review. Eur. J. Med. Chem..

[16] Embuscado M.E. (2015). Spices and herbs: Natural sources of antioxidants—A mini review. J. Funct. Foods.

[17] Eliopoulos C., Markou G., Langousi I., Arapoglou D. (2022). Reintegration of Food Industry By-Products: Potential Applications. Foods.

[18] Abbasi H., Seidavi A., Liu W., Asadpour L. (2015). Investigation on the effect of different levels of dried sweet orange (*Citrus sinensis*) pulp on performance, carcass characteristics and physiological and biochemical parameters in broiler chicken. Saudi J. Biol. Sci..

[19] Jiang X.R., Zhang H.J., Wang J., Wu S.G., Yue H.Y., Lü H.Y., Cui H., Bontempo V., Qi G.H. (2016). Effect of dried tangerine peel extract supplementation on the growth performance and antioxidant status of broiler chicks. Ital. J. Anim. Sci..

[20] Mourão J.L., Pinheiro V.M., Prates J.A.M., Bessa R.J.B., Ferreira L.M.A., Fontes C.M.G.A., Ponte P.I.P. (2008). Effect of dietary dehydrated pasture and citrus pulp on the performance and meat quality of broiler chickens. Poult. Sci..

[21] Mahfuz S., Shang Q., Piao X. (2021). Phenolic compounds as natural feed additives in poultry and swine diets: A review. J. Anim. Sci. Biotechnol..

[22] Pitino R., De Marchi M., Manuelian C.L., Johnson M., Simoni M., Righi F., Tsiplakou E. (2021). Plant Feed Additives as Natural Alternatives to the Use of Synthetic Antioxidant Vitamins on Yield, Quality, and Oxidative Status of Poultry Products: A Review of the Literature of the Last 20 Years. Antioxidants.

[23] Li Y., An M., Wan S., Li Y., Du Y., Zhao Y., Li H., Zhong Q., Sun Z. (2025). Hesperidin enhances broiler growth performance by augmenting gastric acid secretion via the proton pump pathway. Poult. Sci..

[24] Kamboh A.A., Zhu W.Y. (2013). Individual and combined effects of genistein and hesperidin supplementation on meat quality in meat-type broiler chickens. J. Sci. Food Agric..

[25] Li H., Hou Y., Chen J., Wu H., Huang L., Hu J., Zhang Z., Lu Y., Liu X. (2022). Dietary naringin supplementation on laying performance and antioxidant capacity of Three-Yellow breeder hens during the late laying period. Poult. Sci..

[26] Goliomytis M., Kostaki A., Avgoulas G., Lantzouraki D.Z., Siapi E., Zoumpoulakis P., Simitzis P., Deligeorgis S.G. (2018). Dietary supplementation with orange pulp (*Citrus sinensis*) improves egg yolk oxidative stability in laying hens. Anim. Feed. Sci. Technol..

[27] Simitzis P.E., Goliomytis M., Papalexi K., Veneti N., Charismiadou M.A., Kominakis A., Deligeorgis S.G. Effects of flavonoids dietary supplementation on egg yolk antioxidant capacity and cholesterol level. Proceedings of the 65th Annual Meeting of the European Association for Animal Production.

[28] Baira E., Dagla I., Siapi E., Zoumpoulakis P., Simitzis P., Goliomytis M., Deligeorgis S.G., Skaltsounis A.L., Gikas E. (2018). UHPLC–HRMS-based tissue untargeted metabolomics study of naringin and hesperidin after dietary supplementation in chickens. Food Chem..

[29] Baira E., Dagla I., Siapi E., Zoumpoulakis P., Tsarbopoulos A., Simitzis P., Goliomytis M., Deligeorgis S.G., Skaltsounis A.L., Gikas E. (2019). Development of a Validated UHPLC-ESI(-)-HRMS Methodology for the Simultaneous Quantitative Determination of Hesperidin, Hesperetin, Naringin, and Naringenin in Chicken Plasma. Food Anal. Methods.

[30] Baira E., Dagla I., Siapi E., Zoumpoulakis P., Tsarbopoulos A., Simitzis P., Goliomytis M., Deligeorgis S.G., Skaltsounis A.L., Gikas E. (2020). Development and Validation of a UPLC-ESI(-)-MS/MS Methodology for the Simultaneous Quantification of Hesperidin, Naringin, and their Aglycones in Chicken Tissue Samples. J. AOAC Int..

[31] Lohmann Breeders G.H. Lohmann Brown-Classic Layers Management Guide, Cage Housing. https://lohmann-breeders.com/files/downloads/MG/Cage/LB_MG_Cage_LB-Classic_EN.pdf.

[32] Hussein E., Alhotan R.A., Ebrahim A., Selim S. (2023). Unraveling the Potential of Orange Pulp for Improving Laying Rate, Egg Quality, Oxidative Stability, Fatty Acids Composition, and Reproductive Tract Morphology of Laying Hens. Animals.

[33] Abbate J.M., Macrì F., Capparucci F., Iaria C., Briguglio G., Cicero L., Salvo A., Arfuso F., Ieni A., Piccione G. (2020). Administration of Protein Hydrolysates from Anchovy (*Engraulis encrasicolus*) Waste for Twelve Weeks Decreases Metabolic Dysfunction-Associated Fatty Liver Disease Severity in ApoE^−/−^Mice. Animals.

[34] Barbosa C.H., Andrade M.A., Séndon R., Silva A.S., Ramos F., Vilarinho F., Khwaldia K., Barbosa-Pereira L. (2021). Industrial fruits by-products and their antioxidant profile: Can they be exploited for industrial food applications?. Foods.

[35] Chen J., Xia P. (2024). Health effects of synthetic additives and the substitution potential of plant-based additives. Food Res. Int..

[36] Zuñiga-Martínez B.S., Domínguez-Avila J.A., Robles-Sánchez R.M., Ayala-Zavala J.F., Villegas-Ochoa M.A., González-Aguilar G.A. (2022). Agro-Industrial Fruit Byproducts as Health-Promoting Ingredients Used to Supplement Baked Food Products. Foods.

[37] Cuchillo-Hilario M., Fournier-Ramírez M.-I., Díaz Martínez M., Montaño Benavides S., Calvo-Carrillo M.-C., Carrillo Domínguez S., Carranco-Jáuregui M.-E., Hernández-Rodríguez E., Mora-Pérez P., Cruz-Martínez Y.R. (2024). Animal Food Products to Support Human Nutrition and to Boost Human Health: The Potential of Feedstuffs Resources and Their Metabolites as Health-Promoters. Metabolites.

[38] Siwach A., Saini S., Giri A., Khatri P., Kuhad R.C., Kumar A. (2025). Sustainable poultry feed formulations from fruit and vegetable residues for advancing animal health. Bioresour. Technol. Rep..

[39] Chen X.M., Tait A.R., Kitts D.D. (2017). Flavonoid composition of orange peel and its association with antioxidant and anti-inflammatory activities. Food Chem..

[40] Vlaicu P.A., Untea A.E., Panaite T.D., Turcu R.P. (2020). Effect of dietary orange and grapefruit peel on growth performance, health status, meat quality and intestinal microflora of broiler chickens. Italalian J. Anim. Sci..

[41] Zoidis E., Simitzis P., Kampantais D., Katsoulas P., Pappas A.C., Papadomichelakis G., Goliomytis M. (2022). Dietary orange pulp and organic selenium effects on growth performance, meat quality, fatty acid profile, and oxidative stability parameters of broiler chickens. Sustainability.

[42] Nazok A., Rezaei M., Sayyahzadeh H. (2010). Effect of different levels of dried citrus pulp on performance, egg quality, and blood parameters of laying hens in early phase of production. Trop. Anim. Health Prod..

[43] Ferrali M., Signorini C., Caciotti B., Sugherini L., Ciccoli L., Giachetti D., Comporti M. (1997). Protection against oxidative damage of erythrocyte membrane by the flavonoid quercetin and its relation to iron chelating activity. FEBS Lett..

[44] Kuşi M., Becer E., Vatansever H.S. (2024). Basic approach on the protective effects of hesperidin and naringin in Alzheimer’s disease. Nutr. Neurosci..

[45] Siddique S., Firdous S., Durrani A.I., Khan S.J., Saeed A. (2016). Hesperidin, a citrus flavonoid, increases the bioavailability of micronutrients of *Gallus domesticus* (chicken) eggshell: In Vitro study. Chem. Speciat. Bioavailab..

[46] Goff J.P. (2018). Mineral absorption mechanisms, mineral interactions that affect acid–base and antioxidant status, and diet considerations to improve mineral status. J. Dairy Sci..

[47] Li J., Lu H., Liu J., Hong H., Yan C. (2015). The influence of flavonoid amendment on the absorption of cadmium in Avicennia marina roots. Ecotoxicol. Environ. Saf..

[48] Xiao L., Luo G., Tang Y., Yao P. (2018). Quercetin and iron metabolism: What we know and what we need to know. Food Chem. Toxicol..

[49] Jabeen E., Janjua N.K., Ahmed S., Murtaza I., Ali T., Hameed S. (2017). Radical scavenging propensity of Cu^2+^, Fe^3+^ complexes of flavonoids and in-vivo radical scavenging by Fe^3+^-primuletin. Spectrochim. Acta Part A.

[50] Jaccob A.A., Hussain S.A., Hussain S.A. (2012). Effects of long-term use of flavonoids on the absorption and tissue distribution of orally administered doses of trace elements in rats. Pharmacol. Pharm..

[51] Surai P.F. (2014). Polyphenol compounds in the chicken/animal diet: From the past to the future. J. Anim. Physiol. Animal Nutr..

[52] Fedenko V.S., Landi M., Shemet S.A. (2022). Metallophenolomics: A Novel Integrated Approach to Study Complexation of Plant Phenolics with Metal/Metalloid Ions. Int. J. Mol. Sci..

